# Impact of pre‐stenting and bladder dranaige on intrapelvic pressure during retrograde intrarenal surgery

**DOI:** 10.1002/bco2.490

**Published:** 2025-02-04

**Authors:** Sezgin Yeni, Hakan Kilicarslan, Gokhan Ocakoglu, Burhan Coskun, Mehmet Cagatay Cicek, Kadir Omur Gunseren, Ismet Yavascaoglu, Onur Kaygisiz

**Affiliations:** ^1^ Mudanya University Bursa Turkey; ^2^ Medical Faculty, Urology Department Bursa Uludag University Bursa Turkey; ^3^ Medical Faculty, Biostatistics Department Bursa Uludag University Bursa Turkey

**Keywords:** double J stent catheter, Intrapelvic pressure, laser stone lithotripsy, RIRS, Uretral bladder catheter

## Abstract

**Purpose:**

This study aims to assess the effect of pre‐stenting and bladder drainage on intrapelvic pressure (IP) during Retrograde Intrarenal Surgery (RIRS).

**Methods:**

Eighty‐five consecutive patients were prospectively enrolled and meticulously recorded in a data form. Forty‐two patients meeting the inclusion criteria after applying exclusion factors. The patients were divided into two groups: Group 1 (21 patients with preoperative JJ stents) and Group 2 (21 patients without preoperative JJ stents). IP was measured during RIRS, and the impact of various factors, including pre‐stenting, bladder drainage and hydronephrosis (HN) grade, on IP was analysed through univariate and multiple linear regression.

**Results:**

The perioperative mean highest IP (78 ± 18.2 mmHg vs. 110 ± 23.9 mmHg), median lowest IP (29 mmHg vs. 42 mmHg) and median overall IP (41 mmHg vs. 69 mmHg) were significantly lower in Group 1 compared to Group 2 (all p < 0.001). Multivariate analysis showed that pre‐stenting and mild HN (Grade 0–1) were independent predictors of reduced IP.

**Conclusion:**

Pre‐stenting led to a significant reduction in IP during RIRS, likely due to passive ureteral dilation. Additionally, bladder drainage with urethral catheter further decreased IP. These findings suggest that pre‐stenting and bladder drainage should be considered as strategies to reduce IP during RIRS, potentially improving surgical outcomes.

## 1 INTRODUCTION

Retrograde intrarenal surgery (RIRS) has seen a growing utilization in the last two decades for the treatment of kidney stones.^1^, ^2^ According to the guidelines of the European Association of Urology (EAU), shock wave lithotripsy (SWL) and RIRS are recommended as the primary options for kidney stones with a diameter of <2 cm.^3^

In recent years, the investigation of intrapelvic pressure (IP) has become a more frequently studied aspect in minimally invasive kidney stone procedures.^4^ It has been demonstrated that IP exceeding 30 mmHg for more than 50 seconds in percutaneous nephrolithotomy (PNL) is a risk factor for postoperative fever.^5^ Elevated IP has the potential to contribute to complications. In a comprehensive review encompassing studies conducted in both in vitro and in vivo settings, it was observed that IP was higher in RIRS than in PNL.^6^ Despite the higher IP during RIRS compared to PNL, infective complication rates were found to be similar for both surgical methods.^6^

It was demonstrated in a cadaver kidney model during a RIRS study that increasing the access sheath diameter led to an increase in the rate of fluid drainage and a decrease in IP.^7^ Placement of a JJ stent before RIRS increased the diameter of the ureter.^8^ Therefore, it can be predicted that the drainage rate of intrapelvic fluid will increase, and IP will decrease during RIRS as a result of passive ureteral dilatation with pre‐stenting.

The EAU guideline does not provide a specific recommendation for bladder decompression during RIRS.^3^ In an experimental study, it was demonstrated that the use of a urethral catheter for bladder decompression significantly reduces IP during RIRS.^8^ However, according to our knowledge, there is currently no clinical study demonstrating the impact of bladder drainage on IP during RIRS.

To our knowledge, there is no study investigating the impact of pre‐stenting on IP during RIRS. Our primary hypothesis in this study is that pre‐stenting will decrease IP through passive ureteral dilatation during RIRS. The secondary hypothesis is that perioperative bladder drainage during RIRS will also reduce IP.

## 2 MATERIALS AND METHODS

Between September 2, 2019 and October 30, 2021, a total of 85 consecutive patients who underwent retrograde intrarenal surgery (RIRS) were initially enrolled. Following this, exclusion criteria were applied, and the data, along with intrapelvic pressures, for the remaining patients were prospectively measured during RIRS and recorded in a data form.

Randomization was not performed in the study. During the pandemic period, only cancer surgeries were allowed in our operating room. Therefore, 4,7Fr 26 cm DJ stents were placed for pain palliation in stone patients who came to the emergency room. These patients constituted group‐1 and RIRS was applied to these patients approximately 6 weeks after stent placement when elective conditions were met. Intrapelvic pressure data recorded in a data form during surgery were examined retrospectively.

**FIGURE 1:**
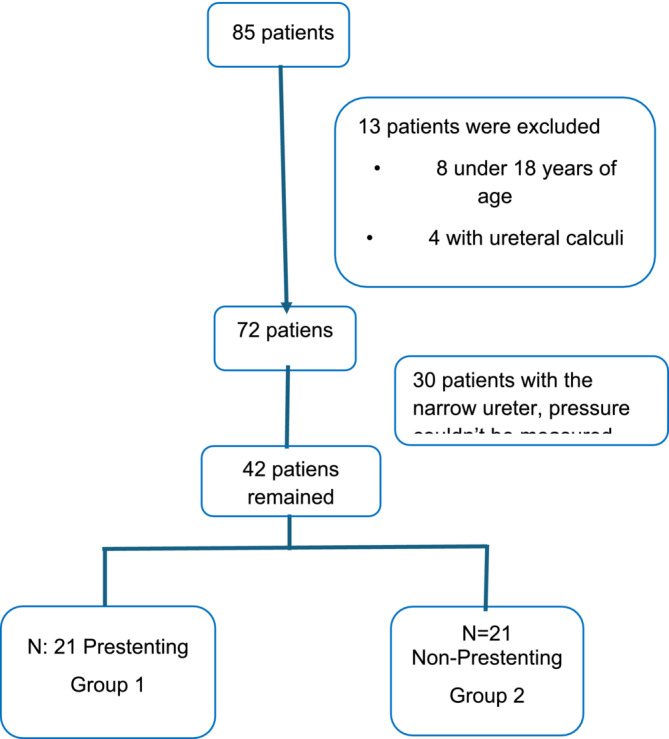
Exclusion criteria chart.

In 30 non‐pre‐stenting patients and none of the pre‐stenting patients, a catheter could not be placed in the kidney due to ureteral stenosis, so IP measurement could not be performed. After excluding patients younger than 18 years of age (n = 8), those with ureteral stones (n = 4) and skeletal anomalies (n = 1), including those for whom pressure could not be measured (n = 30), a total of 42 patients remained in the study. While 21 patients with preoperative JJ stenting constituted Group‐1, 21 patients without preoperative JJ stent constituted Group‐2 **(Figure** **1** **)**.

Stone characteristics and hydronephrosis (HN) grade were evaluated with non‐contrast computed tomography (NCCT) in all patients. HN grading is classified as 0–1–2‐3‐4. In this study, those with SFU grade 0–1 were evaluated as mild HN, and those with 2–3‐4 as severe HN.

Demographic data such as age, gender, body mass index (BMI), HN grade and stone characteristics (location, side, size, density) were recorded. Perioperative and postoperative outcomes (operative time, stone‐free status, complications) and IP results were recorded.

### 2.1 Surgical technique and Intrapelvic pressure measurement

#### 2.1.1 Pressure measurement calibration

In our study, the anaesthesia monitor was calibrated regularly every week. Therefore, we believe the pressure measurement results from this device are accurate. Its accuracy was further confirmed by measuring blood pressure in other patients throughout the day using the same device. Prior to the procedure, we began administering physiological saline with a syringe before connecting the arterial transducer line to the ureter catheter. We observed that when we infused fluid into the catheter with the syringe, the pressure increased and when we stopped, the pressure decreased. This allowed us to verify the accuracy of the pressure measurement system prior to the RIRS procedure.

Under general anaesthesia and the lithotomy position, the height of the irrigation fluid was adjusted to be 60 cm above the patient's kidney level. Then an atraumatic soft sensor guide (Boston Scientific™, 0,035 in. ×150 cm) and A 3F 70 cm 0.018″ open‐ended ureteral catheter was placed with a 22F cystoscope. 2 cc of opaque solution was administered through the catheter to check the proximal end of the catheter was in the renal pelvis under x‐ray fluoroscopy. Then the distal end of the ureteral catheter was connected to the anaesthesia monitor with the arterial transducer. IP was recorded as mmHg once per minute. The median value of the first measured pressures ​was recorded as the control pressure (Pcontrol = Pc).

After the ureteral catheter and sensor guide were placed in the kidney with the help of the cystoscope, the cystoscope was removed without emptying the bladder. A suprapubic physical examination was performed every 2 minutes to monitor bladder fullness during RIRS. The physical examination revealed that the bladder was full at the 5th minute, and intrapelvic pressures did not increase after that.

The median IP during the mapping process with 7.5F flexible ureterorenoscope (Flex X2 Karl Storz™) was recorded as the inlet pressure (Pinlet). Then, 273 nm laser probe was inserted, and laser lithotripsy was started. We did not use a basket catheter in this study because none of the patients required it for stone repositioning. Lithotripsy was performed using fragmentation and dusting techniques. A urethral catheter was placed 15 minutes after the lithotripsy, and bladder drainage was performed. The mean IP was recorded as full bladder (Pbladderfull = Pbf) in the first 15 minutes and empty bladder (Pbladderempty = Pbe) after the 15th minute. The mean pressure decrease after urethral catheterization was calculated as decreased pressure (Pdec = Pbf‐Pbe).

All IP measured during lithotripsy was recorded, and the median of all IP was called IPall (Pall), the highest IP (Phighest = Ph) as a result of squeezing the water pump to improve the quality of the image, lowest IP was recorded as Plowest (Pl).

### 2.2 Statistical analysis

The data of the groups were evaluated using IBM SPSS Statistics 23.0. The normality test of continuous data was performed using the Shapiro–Wilk test. Normally distributed continuous data were compared with the Student‐T test and presented as mean±SD. Non‐normally distributed continuous data were compared with the Mann–Whitney U test and presented as median (minimum‐maximum). Nominal data were compared with the chi‐square test and Fisher's exact test and presented as numbers or percentages. Multiple linear regression analysis was performed to investigate the effects of factors such as age, gender, BMI, stone size, stone localization, stone density, presence of JJ stent and HN grade on IP. P value <0.05 was considered significant.

## 3 RESULTS

The mean age of the patients was 48,5 ± 14,3. The mean stone size was 252 mm^2^ (25–1100 mm^2^) for Group 1 and 225 mm^2^ (100–1225 mm^2^) for Group 2. There was no significant difference between the groups regarding age, gender, BMI and stone characteristics (location, side, size, density) (Table 1).

**TABLE 1:** Demographic data, stone data and perioperative outcomes of the groups.

		Grup 1 (n = 21)	Grup 2 (n = 21)	P
Age (year)		50,5 ± 14,4	44,9 ± 14,1	0,223^1^
Gender (female)		9 (%42,8)	10 (%47,6)	0,537^2^
BMİ (kg/m^2^)		29 (21,9‐53,2)	28,4 (18,7‐45,2)	0,985^3^
Stone side (left)		13 (%61)	10 (%47)	0,242^2^
HN grades	0–1	20 (%95,2)	14(%66,7)	0,045^2^
	2‐3	1 (%4,8)	7 (%33,3)	
Stone size (mm^2^)		252 (75–1100)	225 (100–1225)	0,940^3^
Stone localization	Solitary‐lower pole	6 (%28,5)	3 (%14,2)	0,381^2^
	Solitary‐out of lower pole	7 (%33,3)	11 (%52,3)	
	Multiple	8 (%38)	7 (%33,3)	
Stone density (HU)		1069 ± 302	951 ± 214	0,166^1^
Operative time (min)		67,1 ± 23	63 ± 18	0,544^1^
Stone‐free rate (no/pct.)		16 (%76)	18 (%85)	0,661^2^
CDC		0	0	1
Peroperative compl.		0	0	1

HU: Hounsfield unit, mm^2^: milimeter square, kg/m^2:^ kilogram/m square).

(CDC: Clavien Dindo Classification, min: minute, no: number pct: percentage, compl: complications)

^1^ : Student's t‐test,

^2^ : Fishers's exact test,

^3^ : Mann–Whitney U test.

The mean operative time was calculated as 67.1 ± 23 min in Group 1 and 63 ± 18 min in Group 2 (p = 0.544). While stone‐free status was achieved in 16 (76%) patients in Group 1 and 18 (85%) patients in Group 2, similarly (p = 0.661). No complication occurred in the patients in both groups (Table 1). Postoperative fever was not observed in the patients in our study; therefore, no comparison could be made between the intrapelvic pressure measurementsGrade‐2 HN was detected in four patients and grade‐3 HN in two patients by CT before stent placement, and grade‐2 HN was detected in only one patient by USG after stent placement in Group 1. Six patients had grade‐2 HN and one patient had grade‐3 HN with CT, and the HN grades were found to be the same as those of CT by USG in Group 2. As a result, severe HN was present in one patient in Group 1 and 7 patients in Group 2 (p = 0.045) (Table 1).

The median Pc and median Pinlet measured before lithotripsy were 7 mmHg (6–9 mmHg), 29 mmHg (18–77 mmHg) in Group 1, 9 mmHg (6–12 mmHg), 41 mmHg (24–71 mmHg) in Group 2, respectively ((p < 0.001), (p = 0.002)) (Table 2).

**TABLE 2:** Intrapelvic pressure outcomes of groups.

	Group 1 (n = 21)	Group 2 (n = 21)	P value
Pc (mmHg)	7 (6–9)	9 (6–12)	<0,001^1^
Pinlet (mmHg)	29 (18–77)	41 (24–71)	0,002^1^
Ph (mmHg)	78 ± 18,2	110 ± 23,9	<0,001^2^
Pl (mmHg)	29 (19–54)	42 (17–65)	0,001^1^
Pall (mmHg)	41 (28–70)	69 (35–96)	<0,001^1^
Pbf (mmHg)	46,7 ± 10,9	70 ± 16,9	<0,001^2^
Pbe (mmHg)	40,2 ± 12	61,3 ± 14,7	<0,001^2^
Pdec (mmHg)	8 (2–22)	8 (1–28)	0,909^1^

Pc: control pressure, Pinlet: inlet pressure, Ph: highest pressure, Pl: lowest pressure,

Pall: all pressure, Pbf: pressure of the full bladder, Pbe: pressure of the empty bladder,

Pdec = decreased pressure, Pinc = increased pressure,

^1^ : Mann–Whitney U test,

^2^ : Student's t‐test

The mean highest IP, median lowest IP and median of all IP measured perioperatively were 78 ± 18.2 mmHg, 29 mmHg (19–54 mmHg), 41 mmHg (28–70 mmHg) in Group 1, and 110 ± 23.9 mmHg, 42 mmHg (17–65 mmHg), 69 mmHg (35–96 mmHg) in Group 2, respectively. ((p < 0.001), (p = 0.001), (p < 0.001)) (Table 2).

The age, gender, BMI, stone size, stone localization and stone density solely did not affect the Pall and Ph in the multiple linear regression analysis evaluating the Pall and Ph. This analysis showed that severe HN (grade2–3) was a predictive factor that increases Pall and Ph (p = 0.001, p = 0.007). In addition, pre‐stenting was found to be an independent predictive factor that significantly decreased the Pall during RIRS (p < 0.001) (Table 3).

**TABLE 3:** Multiple linear regression analysis of the factors that effect the median of all IP.

All pressures (pall)	Coefficient	St. error	T	P value
Constant	59,58	3,18	18,75	<0.001
Group				
Group 2 (reference)	‐	‐	‐	‐
Group 1 (x_1_)	−17,73	4,05	−4,38	<0.001
HN Grade				
Grade 0&1(reference)	‐	‐	‐	‐
Grade 2&3	19,26	5,16	3,73	0,001

n = 42, R^2^ = %55 (F = 25,90, p < 0.001).

HN: Hydronephrosis, St: Standard, Pall = median of all intrapelvic pressures.

IP=Intrapelvic pressure.

The mean Ph was calculated as 78.19 ± 18.24 mmHg in Group 1, and 110.29 ± 23.92 mmHg in Group 2, and the corresponding effect size value was determined as d = 1.51. Using the calculated effect size, the power value obtained from our study, where the type I error was accepted as 5%, was determined as 99%, with a total of n = 42 units. (Table 3).

## 4 DISCUSSION

In this study, the impact of pre‐stenting on IP during RIRS was investigated. Although pre‐stenting is not recommended routinely, the EAU guidelines^3^ indicate that it enhances the stone‐free rate and diminishes the risk of intraoperative complications during RIRS. Pre‐stenting facilitates the passage of the ureteral access sheath or ureterorenoscope and it reduces the risk of possible ureteral damage.^9^, ^10^ However, there is currently a lack of studies in the existing literature that specifically investigate the impact of pre‐stenting on intrapelvic pressure (IP) during Retrograde Intrarenal Surgery (RIRS) based on our current knowledge.

There is no definite recommendation in the guideline regarding the fluid height used when making RIRS. It was observed that the IP increased in direct proportion to the height of the fluid in a cadaver study in which the height of the irrigation fluid was determined as 50 cm, 100 cm and 200 cm.^7^ It was shown that the IP increased by 20–25 mmHg when the height of the irrigation fluid was increased from 60 cm to 90 cm.^8^ For this reason, we preferred a 60 cm water height to be safer during RIRS.

It was shown that the use of a 9.5/11.5 F ureteral access sheath did not reduce IP due to inadequate drainage in ex vivo porcine kidneys.^11^ Although the use of a 12/14 F ureteral access sheath reduces IP, it causes ureteral damage by approximately 50% and a reduction of ureteral blood flow by 50%.^11^, ^12^ According to the above‐mentioned studies, there are some advantages and disadvantages of using a 12/14F ureteral access sheath. Routine access sheath usage is not recommended in the EAU guideline because there is no definite information about the long‐term consequences of potential complications.^3^ Therefore, we did not use access sheaths in this study.

In the current study, IP was less in the pre‐stenting group with regard to the control group. According to Bernoulli's principle of fluids, the velocity of the liquid flowing is inversely proportional to the pressure exerted on the surface.^13^ In a study about RIRS in a cadaver kidney model, it was shown that the drainage rate of fluid increased more than two times, and the IP decreased by more than 50% when a 12/14 F UAS was used instead of a 10/12 F UAS.^7^ In that study, decreasing IP in larger UAS can be explained by causing the increase in intrapelvic velocity with increasing drainage velocity based on Bernoulli's principle.^7^, ^13^ It has been shown that the increase in ureteral diameter in patients who were pre‐stented causes a passive ureteral dilatation.^14^ A study by Croghan et al., investigating in vivo ureteroscopic intrarenal pressure (IRP), found that stents were placed in 28% of patients who underwent surgery, with no significant difference in IRP measurements between those who received stents and those who did not.^15^ The results may differ because only 15 of the 122 procedures were performed without an access sheath, while the others used an access sheath with varying calibrations. We believe that the results are more meaningful in our study since it was specifically designed to assess the effect of the stent, unlike the previous study. In a study investigating passive ureteral dilatation in dogs, a JJ stent was placed in the ureter of dogs two weeks before ureteroscopy, and the median width of the ureteral diameter increased from 1.7 mm (1.3–2.7 mm) to 2.8 mm (2.4–3.1 mm) after passive ureteral dilatation.^16^ In our study, JJ stent caused ureteral dilatation that increase intrapelvic fluid velocity and in this wise IP was reduced. So, the expected beneficial effect of the pre‐stenting on all IP during RIRS was achieved. In accordance with our study, Jung H. et al. reported a decrease in IP by dilating the ureter with endoluminal isoproterenol administration during RIRS.^17^

Severe hydronephrosis was found to be higher in Group 2 than in Group 1 due to the regression of HN after JJ stenting of patients in Group 1. Since the amount of fluid in the intrarenal system is higher in patients with severe HN than with mild HN, in the presence of similar ureteral diameters, the amount of drained fluid from the ureter decreases proportionally. Due to the decreased drainage rate of intrarenal fluid, the intrapelvic (IP) pressure was higher in patients with severe HN than with mild HN, as explained by Bernoulli's principle.^13^ At the same time, the stenting process increases ureteral diameter and enhances the drainage rate of fluid from the kidney, resulting in a decrease in IP pressure. In the multivariate analysis conducted in our study, we demonstrated that prestenting reduces IP pressure regardless of the degree of HN.

In an ureterorenoscopy study in pigs in which the bladder was drained with 10 F urethral catheter, the IP was 85 mmHg when the bladder was empty and the IP increased to 110 mmHg when the bladder was full.^8^ Experimental studies have shown that IP is reduced by the placement of a urethral catheter during the procedure.^8^ In this study, we found that the median IP decreased by 8 mmHg in both groups with the placement of a urethral catheter. Although there is no recommendation for bladder decompression in the EAU guideline, our result shows that bladder drainage should be performed to decrease IP during RIRS.^3^

In light of these findings, both of our H_1_ hypotheses were accepted. The pre‐stenting status would reduce the IP by dilating the ureter, and bladder drainage would reduce the pelvic pressure during RIRS.

The limitation of this study is its retrospective nature. However, consecutive patients were included in the study, and perioperative pressure data were contemporaneously recorded on a data form during the operation. While a difference existed between the groups in terms of hydronephrosis (HN) grades, multifactorial analysis demonstrated that pre‐stenting is an independent predictive factor for reducing intrapelvic pressure (IP) during retrograde intrarenal surgery (RIRS). Furthermore, the homogeneous distribution of the groups enhances the reliability of our study.

Also, this study investigated the use of a ureter catheter inserted into the renal pelvis under fluoroscopy before advancing a flexible ureteroscope. Challenges included difficulty positioning the catheter in the renal calyces and potential contact with calyx walls, which could affect pressure readings. To address this, the study ensured the catheter tip avoided contact with any structures during the procedure.

## 5 CONCLUSION

The pre‐stenting resulted in a decreased IP during RIRS. Additionally, we observed that the placement of a drainage catheter into the bladder during the procedure led to a reduction in IP. We suggest considering pre‐stenting for passive dilation before RIRS and the use of a urethral catheter during the procedure to achieve lower intrapelvic pressure during RIRS.

## AUTHOR CONTRIBUTIONS

**Sezgin Yeni:** Rewiew and editing; conceptualization; data curation and writing. **Hakan Kilicarslan:** Editing and conceptualization. **Gokhan Ocakoglu:** Formal analysis; methodology and editing. **Burhan Coskun:** Conceptualization. **Mehmet Cagatay Cicek:** Data curation. **Kadir Omur Gunseren:** Data curation. **Ismet Yavascaoglu:** Methodology and editing. **Onur Kaygisiz:** Rewiew and editing; conceptualization; software; data curation; writing—original draft.

## CONFLICT OF INTEREST STATEMENT

The authors report no conflicts of interest.
